# What Does It Take to Make an Incel: The Role of Paranoid Thinking, Depression, Anxiety, and Attachment Patterns

**DOI:** 10.1155/2024/5512878

**Published:** 2024-06-21

**Authors:** Lilybeth Fontanesi, Daniela Marchetti, Giulia Cosi, Erika Limoncin, Emmanuele A. Jannini, Maria Cristina Verrocchio, Giacomo Ciocca

**Affiliations:** ^1^Department of Psychological, Health and Territorial Sciences, University G. D'Annunzio of Chieti-Pescara, Chieti, Via dei Vestini 31 66100, Italy; ^2^Department of Human Neuroscience, Sapienza University of Rome, Rome, Viale Regina Elena 00185, Italy; ^3^Section of Sexual Psychopathology, Department of Dynamic and Clinical Psychology and Health Studies, Sapienza University of Rome, Rome, Via dei Marsi 78 00185, Italy; ^4^Chair of Endocrinology and Medical Sexology (ENDOSEX), Department of Systems Medicine, University of Rome Tor Vergata, Rome, Via Montpellier 1 00133, Italy

## Abstract

**Background:**

The term “incel” (involuntary celibate) refers to the members of an online subculture, mainly composed of heterosexual men. These individuals find it difficult or impossible to have a romantic and/or sexual partner, and they express extreme anger and resentment toward women, as they hold them accountable for their “inceldom.” In recent years, online and offline acts of violence have been perpetrated by incels, raising concern at social and political levels. This study aims to understand the personal, psychological, and psychopathological variables that may contribute to developing incel traits.

**Materials and Methods:**

A total of 800 Italian heterosexual cisgender men were recruited by a link to the survey forwarded on social networks. Participants have completed a sociodemographic questionnaire and a set of psychometric tests to assess incels' personality traits, attachment patterns, paranoia, anxiety, and depression.

**Results:**

Positive correlations among incel personality traits and psychopathological symptoms and insecure attachment were found. Hierarchical regression analysis showed the significant role of paranoid thinking, insecure attachment, depression, and anxiety symptoms in the presence of incels' personality traits.

**Conclusion:**

Therefore, the assessment of these psychopathological aspects could help clinicians, parents, and teachers to early identify young males that can be caught in the inceldom and to develop specific intervention programs to prevent violence.

## 1. Introduction

The term *incel* (originally defined as involuntary celibates) is commonly used to refer to an online subculture composed mainly of heterosexual men who perceive themselves unable to have heterosexual and consensual sexual or romantic relationships [1]. Nevertheless, a unique and comprehensive definition of the phenomenon is impossible given the subjective nature of involuntary celibacy. According to some authors, self-declared incel has embraced and integrated their own definition of incel philosophy into their sense of self [2, 3]. Incels have unmet sexual desire, and they perceive themselves as victims of sexual liberation and oppressive feminism [4]; incels call their condition “inceldom” [5]. Members of these online communities share highly misogynistic comments and posts; they celebrate violence against women and threaten social attacks [1].

Incels invoke that society is organized according to a very specific hierarchy based on the evolutionary theory that women select their partners according to the “looks, money, and status theory” (LMS theory) [6]. Indeed, women choose their partners according to short-term and long-term preferences, from which “cad” and “dad” sexual strategies evolved [7, 8]. But, according to this theory, physical attractiveness (especially the signs of “good health,” e.g. perfect teeth, large shoulders, and thick hair) and also intelligence, kindness, irony, and humor (characteristics that may guarantee a high social status or may contribute to economic success) are quite important in woman mating preference. Instead, evolutionary theories and hypotheses are intermingled with personal narratives, sexual double standards, sexist, and misogynistic attitudes in the incelosphere [9].

In a few words, according to incels, young and beautiful women are too sexually selective and only attracted to wealthy and/or handsome men on the top of the social pyramid [10], leaving out men who are at the lowest scale of the social hierarchy, who can aspire only to “ugly” partners [11]. On the top of the pyramid, there are the “Chads” (attractive men) and the “Stacys” (attractive women) who embody their ideals of beauty [11]. The Chads can have as much intercourse as they want, which is why the incels despise and envy their status. The Stacys are the object of incel sexual desire, but they are also considered vacuous, superficial, and manipulative [12]. Briefly, according to the LMS theory, the Stacys are too sexually selective and only attracted to rich and/or handsome men (i.e., the Chads) [10], leaving out incels who are excluded from reproduction [11].

In the incel subculture, references are often made to the “pill jargon,” in particular, “red pill,” “blue pill,” and “black pill.” The *redpilled* have metaphorically swallowed the red pill, choosing the “knowledge,” as in the famous movie Matrix, which means that they “recognize” that society is completely dominated by the misandry of feminism [11].

The *bluepilled* reject genetics, LMS theory, and gynocentric society theory. These individuals are considered “simp” because they sympathize with females to gain their attention. The black pill is considered the extreme version of the red pill; it attributes one's inability to establish a relationship to genetics, as it is impossible for some males to transcend social boundaries [13]. In other words, regardless of any attempt to improve themselves, they would still fail to establish a sexual relationship with a woman [14]. The “pill jargon” is widely used throughout the “Men's Rights Activists” (MRA) blog, in the Alt–Right communities, and generally throughout the “manosphere,” which includes the incels. One of the fundamental tenets of the *manosphere* is the centrality of men's rights and the belief that feminism is dangerous to men's social role [15]. According to their beliefs, female emancipation has contributed to the collapse of Western society, and consequently, men have the full right to take revenge against this culture now too-centered on the concept of misandry, to protect its survival and to re-establish a form of male suprematism [12].

The growth of these online communities is extremely worrying not only because of the messages they propagate but also because of the violence being perpetrated online via hate messaging or forms of cyberbullying which, in recent years, has gradually evolved into physical attacks on groups of people. Since 2014, nearly 60 people in the United States have fallen victim to this type of violence [11] [15], and the phenomenon is rapidly spreading even in the European Union [1]. Elliot Rodger, in 2014, was the first to carry out an actual attack recognized as “incel violence”; he killed six people and injured 14 before killing himself [15]. He published a manifesto before the attack that consisted of 133 pages in which he stated, “all I ever wanted was to love women” (in *My Twisted World* by Rodger [16]). It can be hypothesized that those individuals who share psychological risk factors (e.g., antisocial or paranoid traits) bring together individuals who spend substantial time online in misogynistic and closed communities, who may be more susceptible to participating in groups characterized by extremist ideologies and violence. Halpin and colleagues, in fact, found that incel forum users use of misogynistic terms does not increase with post-frequency, suggesting that misogyny is already a characteristic of the forum members [17]. Others, conversely, who show psychological vulnerability, such as a chronic sense of failure, loneliness, or the lack of real-world relationships and interactions, may be more at risk for self-harming [2].

Recent literature on the incel phenomenon has increased, and studies suggest that incels may have more mental health problems than nonincel men [18]. The results of a study conducted by Speckhard and Ellenberg [19] on 272 self-defined incel show that 64.3% of the sample reported depressive symptoms, 24.6% autism traits, 27.9% posttraumatic stress, 59.6% anxiety symptoms, and 47.8% suicidal ideation. These data were also confirmed by an internal survey (2020) conducted on the major incel forum “incel.co,” according to which 74.1% of the sample suffered from anxiety and 67.5% from depression. It has been proposed that people like incels, with poor mating performance, experience more negative feelings such as loneliness or sadness, and they have reported low life satisfaction [20, 21]. Indeed, according to Sparks et al. [22], the loneliness felt by incels may cause them to lack a key buffer that protects them from the adverse effects of romantic rejection and the failure to acquire a romantic or sexual partner conducive to supporting various forms of misogyny and sexism [23]. Unsatisfied mating performance seems to play a key role in the risk of developing incel ideology. Apostolou and colleagues found that in a cross-cultural database of more than 7,000 men from 14 different countries, the 13% define itself as “involuntary single” [24]. Another aspect of the incel's personality is the feeling of being victimized by society and women who deny them the right to have sex [13]. Incels have shown high levels of the tendency for interpersonal victimhood defined as enduring feelings of being victimized in interpersonal relationships, a construct composed of four factors: a need for recognition, moral elitism, a lack of empathy, and rumination [18]. Somehow, these aspects can be associated with paranoid thinking à la “poor me” thinking [25]. Paranoia, fragile masculinity, partner-lessness, and symptoms of anxiety and depression were also related to various mass shootings and episodes of violence by Internet group members [26] and may represent a common subclinical trait among those whose conduct is based on conspiracy theories. Moreover, other discriminatory and deviant behaviors are associated with paranoia, and in many cases, individuals with paranoid tendencies are characterized by anger and aggressiveness [27]. Another relevant aspect of incel psychology is that they crave a partner, but their negative beliefs about women as well as their repulsion for having romantic relations with less attractive girls based on social stereotypes create an impossible situation for incels and might contribute to their anxiety and isolation [14]. Attachment patterns may help in understanding this deadlock. Incels are reported to have different attachment security patterns from nonincels, and also, they have scored higher than their peers on both anxious and avoidant attachment scales [12, 14, 22, 28]. One result of these attachment insecurities is the “anger of despair,” which is particularly linked to alienation and antisocial behavior [29].

The European Commission published an official document addressing the incel phenomenon [1]. According to the report, in Europe, incel culture has had relatively more difficulty taking root due to progressive social values related to feminism, abortion freedom, and beliefs about premarital sex [1]. However, the movement is gradually growing, and the European countries with the highest number of incels are Germany, the United Kingdom, and Sweden. Following that, the UK Commission for Countering Extremism published a research report by Whittaker et al. [4] addressing the incel problem using the largest sample of incels to date (*N* = 561), highlighting the importance of countering the phenomenon from different perspectives. In the European report, Italy is in fourth place [1]. Indeed, in Italy, there are some very popular forums, including the “redpillatore.org,” where a deep difference between incel and the redpilled is emphasized. According to this forum, being an incel is a condition that someone suffers from despite individual incels and most of the members considering it offensive and degrading to call themselves members of the incel community. The redpilled, conversely, aim to observe the reality for what it is and not for how it is proposed on issues such as survival, reproduction, and the biological diversity between men and women [30]. One of Italy's biggest incel forums is “ilforumdegliincel.forumfree.it,” where members define themselves as “incel” based on their extreme political and social ideologies, an identity not just based on the condition of involuntary celibacy [31]. This means that even when some individuals find a mate, they continue to share the incel ideology and be part of that community. Indeed, what the incels' political ideology appears to aim at, along with some far-right movements, is a return to a family-oriented society with men having more control over women and thus having easier access to them [15]. It can be hypothesized thus that incel culture finds breeding grounds in the Italian male population who identify with the values of patriarchal culture. In Italy, indeed, “traditional” gender roles and conservatism seem to be the factors that most influence negative attitudes and violent behaviors toward women [32]. However, the discourse around the incel community and its members is still little addressed in Italy, and little is known about the psychological characteristics and mental health status of individuals in this category. In 2020, a 21-year-old boy killed with 79 stab wounds a pair of roommates with whom he previously lived. In his diary, many sentences related to loneliness, the lack of a partner, and anger toward specific women. Although the perpetrator never declared himself an incel, his story and writings were shared throughout the incel online communities whose members could be seen resonating with the killer's texts and words [33].

In light of these considerations, the goal of the current study was to observe how psychological and personal variables might be related to incel traits. It was possible to hypothesize that the absence of a romantic or sexual partner, paranoid thinking, anxiety, depression, and dysfunctional attachment patterns together are related to incel personality traits. Incel personality traits may inspire an individual to enjoy online communities of incels and the redpilled even without self-identifying as one of them and to engage in violent conduct toward others or themselves. Following the work of Scaptura and Boyle [34], we used an adapted and validated version of the Incel Trait Scale (ITS) [35] to address incels' traits in a general population of heterosexual Italian males. The rationale behind our study was that incel ideology finds fertile ground where psychological fragilities already exist, exacerbating the need to belong to a group with similar “elitist” characteristics and with the same goals of revenge against females and society. In particular, the concurrence of mental disorders in incidents related to incels and the frequency of self-reported mental health challenges on forums underscore the role of mental health issues in contributing to inceldom, with violence being a potential outcome for some individuals on this path [36].

According to risk assessment theory in predicting violent behavior, to assess the likelihood that an individual will engage in aggressive or harmful actions, it is important to address different factors. Mental health, personality characteristics, and social and relational stressors (e.g., not having a romantic or sexual partner) are some of the most important aspects to consider in order to identify and manage potential risks to public safety and individual well-being [36, 37].

It seems crucial, as highlighted in a qualitative study by Longo [38], to preidentify the personal and psychopathological aspects of those who might take part in the incel community to prevent possible violent behavior these individuals might enact against others or themselves, as also suggested by Lankford [39] with the sexual frustration theory of aggression, violence, and crime. In support of the reasons why society should not ignore the incel problem, Blake and Brooks outlined that there is robust evidence for what is referred to in the evolutionary psychology literature as the “young male syndrome” [40]. This theory refers to the tendency for surplus populations of unpartnered young men to disproportionately harm society and themselves, due to increased status-seeking and risk-taking in pursuit of mates [40].

## 2. Materials and Methods

### 2.1. Participants and Procedures

Survey responses were collected via the online platform Qualtrics. A total sample of 1,350 participants were recruited through a link of the survey forwarded on major social networks (Facebook, Instagram, and X) posted to the profiles of a clinical psychology lab and the researchers involved. The inclusion criteria were being at least 18 years old and identifying as a heterosexual cisgender (i.e., reporting a gender identity matched with one's assigned sex at birth) male. We decided not to include nonheterosexual participants in this study according to previous literature which suggested that incel men are predominantly heterosexual.

Participants completed a sociodemographic questionnaire and a set of self-report measures to assess incels' traits, attachment styles, paranoia traits, anxiety, and depression. Participants were informed about the research's aims and privacy practices and did not receive any financial compensation for their participation in the study. Each participant, therefore, gave their own consent for the study by responding to a specific item on the online platform. The entire protocol was anonymous and approved by the Institutional Review Board of Psychology at the Department of Psychological, Health, and Territorial Sciences at the G. d'Annunzio University of Chieti-Pescara (nr. 21004).

Participants completed a sociodemographic questionnaire investigating nationality, educational status, occupation, religion, political ideology, marital status, and the presence of sexual partners. After removing incomplete protocols (40.7%), the final sample was composed of 800 men aged between 18 and 65 years old (*M* = 29.40, SD = 8.03). Demographic and personal characteristics are reported in Table 1.

### 2.2. Incel Traits

The Incel Trait Scale (ITS) is a self-report questionnaire developed by Scaptura and Boyle [34] by the identity theory paradigm [41]. The ITS measures traits and characteristics associated with incel's personality. The English version of ITS is composed of 20 opposite elements, e.g., from “weak” to “strong,” and the participants had to indicate which word described better themselves. Scaptura and Boyle identify two basic factors: “defeat” (13 items) and “hateful” (seven items). The Italian version, validated by Fontanesi and colleagues [35], was used in this study. Unlike the original version, the Italian adaptation comprised 19 pair of items and a three-factor structure: “failure,” “outcasted,” and “enraged.” The first factor, “failure,” assesses the sense of failure, insecurity, and frustration and is composed of nine items; the second five-item factor is outcasted and is associated with a social and relational sense of rejection and ostracization; the last factor, composed by five items, is enraged evaluating the experience of violence, rage, and resentment toward others [35]. An ITS total score was also computed using the sum of the three subscales. The Cronbach alpha for the three subscales and the total scale was failure *α* = 0.81, outcasted *α* = 0.76, enraged = 0.71, and ITS total score *α* = 0.84.

### 2.3. Paranoia

To assess the symptoms of paranoia, we used the Personality Assessment Inventory, a self-report questionnaire designed to provide an evaluation of personality, the presence of psychopathological characteristics, and indications about treatment and compliance [42, 43]. It is composed of 344 items, evaluated on a four-point Likert scale, organized in 22 nonoverlapping subscales. For the present study, we administered only the Paranoia Scale (PAR), 24 items subdivided into three subscales of hypervigilance, persecution, and resentment, directly assessing distrust and interpersonal hostility. The Cronbach's alpha for the total paranoia scale was *α* = 0.86, for the hypervigilance subscale was *α* = 0.70, for the persecution factor Cronbach's alpha was 71, and for the resentment factor was 0.83.

### 2.4. Anxiety

In this study, the two-item version of the Generalized Anxiety Disorder (GAD-2) by Staples et al. [44] was used to screen anxiety symptoms in our sample. The items are (1) “Feeling nervous, anxious or tense” and (2) “Not being able to stop or control worries.” Participants can express their anxiety levels through a four-point Likert scale; a score ≥3 may indicate the presence of a clinically relevant anxiety disorder.

### 2.5. Depression

The Patient Health Questionnaire – 2 (PHQ – 2) was used to evaluate the presence of depressive symptoms [44]. The items, rated on a four-point Likert scale, are (1) “Little interest or pleasure in doing things” and (2) “Feeling down, depressed, or hopeless.” A score ≥3 may indicate the presence of clinically relevant depressive symptoms.

### 2.6. Attachment Patterns

The Italian version of the Relationship Questionnaire (RQ) [45] was used to assess adult attachment. The RQ is a single-item instrument consisting of four paragraphs, each describing a prototypical attachment pattern in adult peer and romantic relationships. The four attachment styles evaluated are secure, preoccupied, fearful, and dismissing. These patterns are described according to two main dimensions: anxiety defined as a strong need for care and attention from attachment figures and a pervading uncertainty regarding the willingness of attachment figures to be responsive to these needs, and avoidance, a strong discomfort with emotional intimacy and the desire to keep psychological independence. Participants are asked to rate their agreement with each prototype on a seven-point Likert scale.

### 2.7. Statistical Analysis

All statistical analyses were computed using IBM SPSS Statistics version 26.0 [46]. First, mean and standard deviation have been calculated for each variable, and then Pearson correlations have been computed to explore univariate associations between the study variables. Next, four stepwise multiple linear regression analyses were conducted to analyze the single contribution of each variable on the development of ITS total and subscales scores. The ITS total score, failure, outcasted, and enraged factors were entered as dependent variables in the four regressions. In the first step, personal variables such as age, schooling years, and the presence of sexual partners were entered as independent variables, followed by paranoia subscales in the second step, anxiety, and depression in the third step and, lastly, attachment patterns in the fourth step.

## 3. Results and Discussion

Pearson correlation analysis was conducted to analyze relationships between the study variables. Results of the analysis are reported in Table 2, along with means and standard deviations. Remarkably, ITS total scores positively correlated with higher scores on the paranoia scale and subscale, in particular “resentment” (*r* = 0.411, *p*  < 0.01). Resentment, characterized by an external locus of control and bitterness in interpersonal relationships, positively correlated with all dimensions of the ITS, in particular with the subscale “outcast” (*r* = 0.495, *p*  < 0.01). ITS total scores also correlated highly with anxiety and depression symptoms and with insecure attachment patterns (Table 2). Interestingly, the factor “failure” highly correlated with depression (*r* = 0.539, *p*  < 0.01) and anxiety (*r* = 0.456, *p*  < 0.01), while the factor “enraged” had a very low positive significant association with these dimensions.

A series of four stepwise hierarchical regressions were performed using ITS total score and subfactors as dependent variables, and personal variables, paranoia subscales, symptoms of anxiety and depression, and attachment patterns as independent variables.  Table 3 reports the regression analysis on the ITS total score, where the model accounts for 45% of the total variance. After the fourth step low education level, the absence of a sexual partner hypervigilance and resentment anxiety and depression and higher levels of insecure attachment patterns (i.e., preoccupied and fearful) were all significant predictors of higher scores on the trait “incel,” with paranoia subscales adding the largest percentage of variance (*Δ*R^2^ = 0.171, *p*  < 0.001) (Table 3).

To disentangle the contribution of each considered variable in explaining each single ITS dimension, further stepwise hierarchical regressions were performed.  Table 4 reports the stepwise hierarchical regression analysis for the failure subfactor of the ITS. The 46% of variance of the ITS failure score is explained in particular by anxiety and depression symptoms and fearful attachment (Table 4).

Tables 5 and 6 show the hierarchical regression analysis with “outcasted” and “enraged” subfactors as dependent variables. Thirty seven percent of the total variance of “outcast” was explained by the same variables as failure, except for anxiety (*β* = 0.01, n.s.). In contrast with the other two subfactors, the “enraged” trait was mostly predicted by paranoia, in particular hypervigilance and resentment. Also, the avoidant attachment style had an important influence on the development of the enraged trait, as reported in Table 6, accounting for 16% of the total variance.

Overall, the present study investigated the role of personal and psychological variables in the relationship with incel personality traits in a sample of general population males. The aim of the study was to assess personal and psychological risk factors that might combine to inspire an individual to enjoy the incel/redpilled communities. Our findings suggest that the absence of a sexual partner, paranoid thinking, anxiety, and depression, as well as insecure attachment patterns (in particular, fearful attachment), are associated with incel personality traits, related to feeling marginalized by society, excluded from romantic relationships, and feeling anger toward women. Our results cohere with the international literature [4], suggesting that, in general, poor mating performance in men correlates with lower self-satisfaction and symptoms of anxiety and depression [28, 47]. Moreover, according to prior research linking self-perceived mate value to men's self-esteem [48], men, on average, are more misogynistic when they doubt their appeal to women [49] or experience involuntary celibacy [23]. Some research suggests that incels blame themselves for their [2, 28]; others highlight their external locus of control [18]. It is possible, then, that what distinguishes these young men from their single peers is the belief that their condition of loneliness and their sense of failure is someone or something else's fault, particularly women, feminist movements, or social determinism. This mental mechanism might originate in paranoid thinking, such as resentment toward those who rejected them and society itself. Somehow, their condition then becomes an inescapable and predestined failure; hence, anger-related traits are in turn influenced and maintained by the resentment and persecutory thinking typical in paranoia. The resentment component of paranoid thinking is an emotion characterized by feelings of bitterness, an indignation connected to a real or perceived injustice or wrongdoing. It can stem from real experiences of betrayal, humiliation, or unfair treatment. These emotional wounds may fuel paranoid thoughts and contribute to the development of a mistrustful worldview. Our results showed that the three components of ITS are highly influenced by resentment, which is arguably the most influential element of paranoia in the development of incel personality traits, suggesting that an external locus of control and vengeance can be risk factors of violent conduct. Moreover, if paranoid thinking is associated with feelings of depression and anxiety, the suffering of these individuals becomes unbearable. Their vulnerability and hatred can become fertile ground for participation in misogynist and conservative extremist groups online, which offer these young people the opportunity to express their feelings and find commonality in fighting a system that penalizes men and favors women, once they swallow the black pill and become aware of the “evolutionary pattern.” It is possible that individuals with mental health symptoms, such as anxiety and depression, are more likely than others to feel attracted to the incel community where they experience peer acceptance [11]. Simultaneously, it is possible that the experiences within this environment are alienating in respect to mainstream movements but at the same time colored by hopeless rhetoric that aggravates already existing psychological difficulties, as hopelessness, sadness, resentment, and suicidal ideation are a prominent topic of discussion in most of the incel communities [10]. In our study, fearful and preoccupied attachment patterns also played an important role in the scores of incels' traits, in particular, failure and outcasted subtraits, by means that the fear of rejection might create a vicious cycle, promoting isolation and withdrawal from meaningful relationships. This may strengthen incel identity and foster a sense of “brotherhood” in the community, by limiting other members from engaging with the mating market, and mitigate negative feelings related to rejection [2]. What is particularly interesting, however, is how the enraged factor was instead related to avoidant attachment—the “anger of despair” described by Mikulincer—which suggested the presence in avoidant individuals of hostile and negative anger connected to violent acts, alienation, and antisocial behaviors [50]. In this regard, many facets of psychological suffering are associated with insecure attachment styles, fear, and preoccupation [38]. Many studies have demonstrated a strong relationship between fearful and preoccupied attachment styles with subclinical and clinical mental conditions [51, 52]. In the presence of loneliness and a dearth of stable, meaningful relationships, individuals may define violence—both physical and virtual—as a way to find redemption and overcome low self-esteem. As explained by Linder analysis [53], violence could be interpreted as the ultimate reaction to signals of diminished value and unsuccessful efforts to readjust the target.

Moreover, it is noteworthy that the suicidality that pervades the incelosphere could be a response to the perceived ostracism from society [54]. Loneliness can be processed by humans as a catastrophic condition, according to the evolutionary theory, in fact, social isolation, in an ancestral environment, would equate to a death sentence [55]. That might not be the case of the modern environment, but literature on incels highlights how they suffer for the lack of friends and meaningful and supportive relations [12, 18]. Consequently, in association with depression and anxiety [56], the absence of significant relationships might influence the occurrence of suicidal thinking, as reported in many inceldom forums [54].

## 4. Conclusions

Our study suggests that insecure attachment patterns, paranoia, and internalized symptoms, such as anxiety and depression symptoms, influence the development of incel personality traits, which is a condition of social isolation, low self-esteem, and a sense of failure related to the lack of a romantic or sexual partner, common in young men. Clinicians and psychologists should monitor these situations, especially in high schools and colleges, and take care of the mental well-being of these individuals with specific prevention programs directed at both decreasing the social stigma against these people and facilitating their access to psychological therapy. These prevention strategies are fundamental to avoid more problematic situations characterized by deviant and violent behavior or the onset of mental illness.

Early prevention programs in schools should be directed towards achieving two goals: on the one hand, to implementing effective sexual education programs that increase awareness concerning gender equality, respect for sexual rights, sexual consent, and privacy [57]. This might both result in a decrease in stereotypes related to gender bias and prevent misogynistic attitudes and eventual violent conduct, both online and off. On the other hand, a discussion with a health professional in a safe environment about emotional expression and relational difficulties might help these young men come to terms with their fragilities and thus prevent self-harm and suicidal ideation. These programs could prevent these people from seeking comfort and support online through red-pill and black-pill groups that sharpen their distorted thinking and act as triggers that turn despair into violence.

The findings of this study have significance for evolving the empirical exploration of the incel phenomenon. This is especially crucial not only for averting its potential escalation into violence but, more importantly, for promptly identifying the distress of individuals exhibiting incel traits who might be reluctant to seek psychological assistance. The presentation of specific psychological aspects discussed and assessed in this study can help clinicians, parents, and teachers to preidentify young male incel traits and risk factors to prevent mental distress and violent conduct.

## Figures and Tables

**Table 1:** Characteristics of the sample (*n* = 800).

Variables	Frequencies (%)
Nationality	
Italian	789 (98.6)
Foreign	11 (1.4)
School years	
8	23 (2.9)
13	345 (42.1)
16	202 (25.3)
18	164 (20.5)
More than 18	66 (8.2)
Occupation	
Unoccupied (or student)	354 (44.3)
Occupied (employed)	446 (55.8)
Religion	
Catholic or others	312 (39)
Atheist/agnostic	488 (61)
Political ideology	
Conservative (right parties)	133 (16.6)
Moderate	213 (26.6)
Progressive (left parties)	454 (56.8)
Relational status	
Married	91 (11.4)
Single	402 (50.2)
In a relationship	298 (37.3)
Divorced	9 (1.2)
Sexual partner	
More than one sexual partner	93 (11.6)
One regular partner	381 (47.6)
No sexual partner	326 (40.8)

**Table 2:** Pearson correlations, means, and standard deviations of the study variables.

	1	2	3	4	5	6	7	8	9	10	11	12	13	14	15	M	DS
1. Age	—	—	—	—	—	—	—	—	—	—	—	—	—	—	—	29.32	7.94
2. School years	0.163^*∗∗*^	—	—	—	—	—	—	—	—	—	—	—	—	—	—	15.22	2.69
3. ITS failure	−0.133^*∗∗*^	−0.138^*∗∗*^	—	—	—	—	—	—	—	—	—	—	—	—	—	28.78	14.47
4. ITS outcast	−0.044	−0.105^*∗∗*^	0.697^*∗∗*^	—	—	—	—	—	—	—	—	—	—	—	—	15.36	7.098
5. ITS enraged	−0.028	−0.112^*∗∗*^	0.219^*∗∗*^	0.371^*∗∗*^	—	—	—	—	—	—	—	—	—	—	—	11.87	7.06
6. ITS total score	−0.103^*∗∗*^	−0.152^*∗∗*^	0.903^*∗∗*^	0.866^*∗∗*^	0.553^*∗∗*^	—	—	—	—	—	—	—	—	—	—	56.02	23.84
7. PAR hypervigilance	−0.135^*∗∗*^	−0.136^*∗∗*^	0.208^*∗∗*^	0.280^*∗∗*^	0.339^*∗∗*^	0.320^*∗∗*^	—	—	—	—	—	—	—	—	—	14.55	3.48
8. PAR persecution	−0.145^*∗∗*^	−0.100^*∗∗*^	0.138^*∗∗*^	0.226^*∗∗*^	0.284^*∗∗*^	0.243^*∗∗*^	0.526^*∗∗*^	—	—	—	—	—	—	—	—	9.93	3.18
9. PAR resentment	−0.137^*∗∗*^	−0.153^*∗∗*^	0.319^*∗∗*^	0.389^*∗∗*^	0.295^*∗∗*^	0.411^*∗∗*^	0.493^*∗∗*^	0.496^*∗∗*^	—	—	—	—	—	—	—	6.34	2.69
10. PAR total score	−0.170^*∗∗*^	−0.157^*∗∗*^	0.264^*∗∗*^	0.357^*∗∗*^	0.375^*∗∗*^	0.390^*∗∗*^	0.845^*∗∗*^	0.827^*∗∗*^	0.780^*∗∗*^	—	—	—	—	—	—	30.82	7.67
11. GAD-2 anxiety	−0.172^*∗∗*^	−0.069	0.456^*∗∗*^	0.284^*∗∗*^	0.152^*∗∗*^	0.416^*∗∗*^	0.263^*∗∗*^	0.153^*∗∗*^	0.297^*∗∗*^	0.287^*∗∗*^	—	—	—	—	—	4.57	1.65
12. PHQ-2 depression	−0.133^*∗∗*^	−0.099^*∗∗*^	0.539^*∗∗*^	0.385^*∗∗*^	0.137^*∗∗*^	0.495^*∗∗*^	0.233^*∗∗*^	0.192^*∗∗*^	0.349^*∗∗*^	0.308^*∗∗*^	0.598^*∗∗*^	—	—	—	—	4.17	1.63
13. RQ secure	0.034	0.043	−0.421^*∗∗*^	−0.402^*∗∗*^	−0.045	−0.402^*∗∗*^	−0.141^*∗∗*^	−0.079^*∗*^	−0.197^*∗∗*^	−0.166^*∗∗*^	−0.178^*∗∗*^	−0.250^*∗∗*^	—	—	—	3.34	1.71
14. RQ preocuppied	−0.134^*∗∗*^	−0.032	0.372^*∗∗*^	0.356^*∗∗*^	0.197^*∗∗*^	0.403^*∗∗*^	0.334^*∗∗*^	0.236^*∗∗*^	0.315^*∗∗*^	0.360^*∗∗*^	0.271^*∗∗*^	0.333^*∗∗*^	−0.364^*∗∗*^	—	—	3.62	1.88
15. RQ fearful	−0.199^*∗∗*^	−0.080^*∗*^	0.401^*∗∗*^	0.405^*∗∗*^	0.145^*∗∗*^	0.421^*∗∗*^	0.229^*∗∗*^	0.251^*∗∗*^	0.301^*∗∗*^	0.314^*∗∗*^	0.281^*∗∗*^	0.288^*∗∗*^	−0.223^*∗∗*^	0.399^*∗∗*^	—	3.02	1.78
16. RQ avoidant	−0.019	0.004	−0.033	−0.002	0.091^*∗∗*^	0.006	0.062	0.013	−0.044	0.018	−0.073^*∗*^	−0.029	−0.097^*∗∗*^	0.161^*∗∗*^	−0.056	4.13	1.77

*Note*. *⁣*^*∗*^*p* < 0.05*; *⁣*^*∗∗*^*p*  < 0.01*.

**Table 3:** Stepwise linear hierarchical regression with Incel Trait Scale total score as dependent variable.

Variables	Step 1			Step 2			Step 3			Step 4		
	*B*	*SEB*	*β*	*B*	*SEB*	*β*	*B*	*SEB*	*β*	*B*	*SEB*	*β*
Personal variables												
Age	−0.18	0.10	−0.06	−0.01	0.10	−0.002	0.10	0.09	0.03	0.15	0.08	0.05
Schooling years	−2.64	0.74	−0.12^*∗∗∗*^	−1.30	0.68	−0.06	−1.19	0.62	−0.05	−1.36	0.58	−0.06^*∗*^
Sexual partner	−6.72	1.25	−0.19^*∗∗∗*^	−7.32	1.13	−0.20^*∗∗∗*^	−5.80^*∗∗∗*^	1.05	−0.16	−3.21	1.02	−0.09^*∗∗∗*^
Paranoia subscales												
Hypervigilance	—	—	—	1.04	0.26	0.15^*∗∗∗*^	0.72^*∗∗*^	0.24	0.10	0.49	0.23	0.07^*∗*^
Persecution	—	—	—	0.05	0.29	0.01	0.13	0.27	0.02	0.01	0.25	0.001
Resentment	—	—	—	2.86	0.34	0.32^*∗∗∗*^	1.80^*∗∗∗*^	0.32	0.20	1.27	0.3	0.14^*∗∗∗*^
Symptoms												
Anxiety	—	—	—	—	—	—	2.14^*∗∗∗*^	0.52	0.15	1.67	0.50	0.12^*∗∗∗*^
Depression	—	—	—	—	—	—	4.17^*∗∗∗*^	0.54	0.28	3.22	0.51	0.22^*∗∗∗*^
Attachment styles												
Secure	—	—	—	—	—	—	—	—	—	−2.81	0.41	−0.21^*∗∗∗*^
Preoccupied	—	—	—	—	—	—	—	—	—	0.99	0.41	0.08^*∗*^
Fearful	—	—	—	—	—	—	—	—	—	2.40	0.41	0.18^*∗∗∗*^
Avoidant	—	—	—	—	—	—	—	—	—	0.04	0.37	0.001
*R*	—	0.252	—	—	0.484	—	—	0.602	—	—	0.670	—
*R^2^*	—	0.064	—	—	0.234	—	—	0.362	—	—	0.449	—
*ΔR^2^*	—	0.064	—	—	-0.171	—	—	0.128	—	—	0.087	—
*F*	—	18.01^*∗∗∗*^	—	—	40.48^*∗∗∗*^	—	—	56.16^*∗∗∗*^	—	—	53.53^*∗∗∗*^	—

Note. Sexual partner: 1 = having a sexual partner, 0 = not having a sexual partner; *⁣*^*∗*^ = *p*  > 0.05; *⁣*^*∗∗*^*p*  > 0.01; *⁣*^*∗∗∗*^*p*  < 0.001.

**Table 4:** Stepwise linear hierarchical regression with failure factor as dependent variable.

Variables	Step 1			Step 2			Step 3			Step 4		
	*B*	*SEB*	*β*	*B*	*SEB*	*β*	*B*	*SEB*	*β*	*B*	*SEB*	*β*
Personal variables												
Age	−0.12	0.06	−0.07	−0.05	0.06	−0.03	0.01	0.05	0.01	0.01	0.05	0.01^*∗*^
Schooling years	−1.25	0.44	−0.10^*∗∗*^	−0.69	0.43	−0.05^*∗∗∗*^	−0.64	0.37	−0.05	−0.77	0.35	−0.06
Sexual partner	−7.03	1.02	−0.24^*∗∗∗*^	−7.07	0.97	−0.24	−5.17	0.87	−0.17	−2.18	0.85	−0.07^*∗∗*^
Paranoia subscales												
Hypervigilance	—	—	—	0.33	0.16	0.08^*∗*^	0.09	0.15	0.02	−0.03	0.14	−0.01
Persecution	—	—	—	−0.24	0.18	−0.05	−0.18	0.16	−0.04	−0.24	0.15	−0.05
Resentment	—	—	—	1.53	0.21	0.28^*∗∗∗*^	0.74	0.19	0.14	0.40	0.18	0.07^*∗∗∗*^
Symptoms												
Anxiety	—	—	—	—	—	—	1.70	0.31	0.19^*∗∗∗*^	1.37	0.29	0.16^*∗∗∗*^
Depression	—	—	—	—	—	—	3.01	0.32	0.34^*∗∗∗*^	2.51	0.31	0.28^*∗∗∗*^
Attachment styles												
Secure	—	—	—	—	—	—	—	—	—	−1.98	0.24	−0.23^*∗∗∗*^
Preoccupied	—	—	—	—	—	—	—	—	—	0.49	0.25	0.06
Fearful	—	—	—	—	—	—	—	—	—	1.34	0.25	0.17^*∗∗∗*^
Avoidant	—	—	—	—	—	—	—	—	—	−0.29	0.22	−0.04
*R*	—	0.293	—	—	0.419	—	—	0.607	—	—	0.676	—
*R^2^*	—	0.086	—	—	0.176	—	—	0.368	—	—	0.456	—
*ΔR^2^*	—	0.086	—	—	0.090	—	—	0.192	—	—	0.088	—
*F*	—	24.90^*∗∗∗*^	—	—	28.21^*∗∗∗*^	—	—	57.55^*∗∗∗*^	—	—	55.05^*∗∗∗*^	—

*Note*. Sexual partner: 1 = having a sexual partner, 0 = not having a sexual partner; *⁣*^*∗*^ = *p*  > 0.05; *⁣*^*∗∗*^*p*  > 0.01; *⁣*^*∗∗∗*^*p*  < 0.001.

**Table 5:** Stepwise linear hierarchical regression with outcast factor as dependent variable.

Variables	Step 1			Step 2			Step 3			Step 4		
	*B*	*SEB*	*β*	*B*	*SEB*	*β*	*B*	*SEB*	*β*	*B*	*SEB*	*β*
Personal variables												
Age	0.03	0.03	0.02	0.08	0.033	0.08^*∗*^	0.09	0.03	0.09^*∗∗*^	0.10	0.03	0.10^*∗∗∗*^
Schooling years	−0.55	0.25	−0.08*⁣*^*∗*^	−0.13	0.227	−0.02	−0.11	0.22	−0.01	−0.17	0.21	−0.02
Sexual partner	−4.33	0.57	−0.27*⁣*^*∗∗∗*^	−4.43	0.519	−0.27*⁣*^*∗∗∗*^	−3.84	0.51	−0.24*⁣*^*∗∗∗*^	−2.15	0.50	−0.13*⁣*^*∗∗∗*^
Paranoia subscales												
Hypervigilance	—	—	—	0.27	0.09	0.12^*∗∗*^	0.21	0.09	0.09^*∗*^	0.15	0.08	0.06
Persecution	—	—	—	0.04	0.09	0.01	0.05	0.09	0.02	0.01	0.09	0.005
Resentment	—	—	—	0.95	0.11	0.32^*∗∗∗*^	0.73	0.11	0.24^*∗∗∗*^	0.54	0.11	0.18^*∗∗∗*^
Symptoms												
Anxiety	—	—	—	—	—	—	0.26	0.18	0.05	0.07	0.17	0.01
Depression	—	—	—	—	—	—	1.02	0.19	0.21^*∗∗∗*^	0.75	0.18	0.15^*∗∗∗*^
Attachment styles												
Secure	—	—	—	—	—	—	—	—	—	−1.04	0.14	−0.22*⁣*^*∗∗∗*^
Preoccupied	—	—	—	—	—	—	—	—	—	0.19	0.15	0.04
Fearful	—	—	—	—	—	—	—	—	—	0.90	0.15	0.20^*∗∗∗*^
Avoidant	—	—	—	—	—	—	—	—	—	−0.06	0.13	−0.01
*R*	—	0.282	—	—	0.484	—	—	—	0.533	—	0.612	—
*R^2^*	—	0.079	—	—	0.234	—	—	—	0.284	—	0.375	—
*ΔR^2^*	—	0.079	—	—	0.155	—	—	—	0.050	—	0.091	—
*F*	—	22.88^*∗∗∗*^	—	—	40.42^*∗∗∗*^	—	—	—	39.18^*∗∗∗*^	—	39.33^*∗∗∗*^	—

*Note*. Sexual partner: 1 = having a sexual partner, 0 = not having a sexual partner; *⁣*^*∗*^ = *p*  > 0.05; *⁣*^*∗∗*^*p*  > 0.01; *⁣*^*∗∗∗*^*p*  < 0.001.

**Table 6:** Stepwise linear hierarchical regression with enraged factor as dependent variable.

Variables	Step 1			Step 2			Step 3			Step 4		
	*B*	*SEB*	*β*	*B*	*SEB*	*β*	*B*	*SEB*	*β*	*B*	*SEB*	*β*
Personal variables												
Age	−0.007	0.03	−0.01	0.04	0.03	0.05	0.05	0.03	0.05	0.05	0.03	0.06
Schooling years	−0.69	0.22	−0.11*⁣*^*∗∗*^	−0.35	0.21	−0.06	−0.36	0.21	−0.06	−0.39	0.21	−0.06
Sexual partner	−0.15	0.52	−0.01	−0.32	0.48	−0.02	−0.31	0.49	−0.02	−0.23	0.51	−0.01
Paranoia subscales												
Hypervigilance	—	—	—	0.44	0.08	0.22^*∗∗∗*^	0.42	0.08	0.21^*∗∗∗*^	0.38	0.08	0.19^*∗∗∗*^
Persecution	—	—	—	0.23	0.09	0.10^*∗∗*^	0.24	0.09	0.11^*∗∗*^	0.22	0.09	0.10^*∗∗*^
Resentment	—	—	—	0.35	0.11	0.13^*∗∗∗*^	0.33	0.11	0.12^*∗∗*^	0.34	0.11	0.13^*∗∗*^
Symptoms												
Anxiety	—	—	—	—	—	—	0.23	0.18	0.05	0.24	0.18	0.06
Depression	—	—	—	—	—	—	−0.04	0.18	−0.01	−0.07	0.19	−0.01
Attachment styles												
Secure	—	—	—	—	—	—	—	—	—	0.26	0.15	0.06
Preoccupied	—	—	—	—	—	—	—	—	—	0.23	0.15	0.06
Fearful	—	—	—	—	—	—	—	—	—	0.09	0.15	0.02
Avoidant	—	—	—	—	—	—	—	—	—	0.34	0.13	0.09^*∗*^
*R*	—	0.113	—	—	0.385	—	—	0.388	—	—	0.404	—
*R^2^*	—	0.013	—	—	0.148	—	—	0.150	—	—	0.163	—
*ΔR^2^*	—	0.013	—	—	0.136	—	—	0.002	—	—	0.013	—
*F*	—	3.41^*∗∗∗*^	—	—	40.42^*∗∗∗*^	—	—	39.18^*∗∗∗*^	—	—	39.33^*∗∗∗*^	—

*Note*. Sexual partner: 1 = having a sexual partner, 0 = not having a sexual partner; *⁣*^*∗*^*p*  > 0.05; *⁣*^*∗∗*^*p*  > 0.01; *⁣*^*∗∗∗*^*p*  < 0.001.

## Data Availability

The data presented in this study are available from the corresponding author upon reasonable request.

## Additional Points

*Limitations and Future Directions*. The present study had certain limitations. First, we did not directly select self-declared incel. It is highly likely that given the presence of the survey on various social networks, some of the participants identified themselves as incel; however, future studies should replicate these results with a sample of people who define themselves as incel. Second, we did not directly consider aspects related to misogyny and sexism (i.e., malevolent or benevolent sexism or gender discrimination), so it would be appropriate to analyze the relationships between the variables we analyzed and these two constructs. Moreover, future research should further investigate the relationship between poor mental health, violence, and other specific conditions such as, for example, autism, since previous literature has identified a correlation between ASD harmful attitudes and belief in incel community [4]. Also, we did not evaluate aspects related to political ideologies and belonging to incel communities in this study, as most of the participants declared themselves to be “left-wing,” so it would be interesting to study more heterogeneous groups to assess this relationship already documented in the international literature [4].
